# Lessons from a large trauma center: impact of blunt chest trauma in polytrauma patients—still a relevant problem?

**DOI:** 10.1186/s13049-017-0384-y

**Published:** 2017-04-20

**Authors:** Konstantina Chrysou, Gabriel Halat, Beatrix Hoksch, Ralph A. Schmid, Gregor J. Kocher

**Affiliations:** Division of General Thoracic Surgery, Inselspital, Bern University Hospital, University of Bern, CH – 3010 Bern, Switzerland

## Abstract

**Background:**

Thoracic trauma is the third most common cause of death after abdominal injury and head trauma in polytrauma patients. The purpose of this study was to investigate epidemiological data, treatment and outcome of polytrauma patients with blunt chest trauma in order to help improve management, prevent complications and decrease polytrauma patients’ mortality.

**Methods:**

In this retrospective study we included all polytrauma patients with blunt chest trauma admitted to our tertiary care center emergency department for a 2-year period, from June 2012 until May 2014. Data collection included details of treatment and outcome. Patients with chest trauma and Injury Severity Score (ISS) ≥18 and Abbreviated Injury Scale (AIS) >2 in more than one body region were included.

**Results:**

A total of 110 polytrauma patients with blunt chest injury were evaluated. 82 of them were males and median age was 48.5 years. Car accidents, falls from a height and motorbike accidents were the most common causes (>75%) for blunt chest trauma. Rib fractures, pneumothorax and pulmonary contusion were the most common chest injuries. Most patients (64.5%) sustained a serious chest injury (AIS_thorax_ 3), 19.1% a severe chest injury (AIS_thorax_ 4) and 15.5% a moderate chest injury (AIS_thorax_ 2). 90% of patients with blunt chest trauma were treated conservatively. Chest tube insertion was indicated in 54.5% of patients. The need for chest tube was significantly higher among the AIS_thorax_ 4 group in comparison to the AIS groups 3 and 2 (*p* < 0.001). Also, admission to the ICU was directly related to the severity of the AIS_thorax_ (*p* < 0.001). The severity of chest trauma did not correlate with ICU length of stay, intubation days, complications or mortality.

**Conclusion:**

Although 84.5% of patients suffered from serious or even severe chest injury, neither in the conservative nor in the surgically treated group a significant impact of injury severity on ICU stay, intubation days, complications or mortality was observed. AIS_thorax_ was only related to the rate of chest tube insertions and ICU admission. Management with early chest tube insertion when necessary, pain control and chest physiotherapy resulted in good outcome in the majority of patients.

## Background

Despite global efforts to reduce human accidents, even today, the number of polytrauma patients admitted to hospitals remains high with around 5.8 million deaths worldwide attributed to traumatic injuries every year [1, 2]. The differences in the severity and complexity of these injuries constitute a challenging issue for clinicians around the world and therefore advanced health care methods are needed in order to reduce morbidity and mortality rates.

Thoracic trauma constitutes the third most common cause of death after abdominal injury and head trauma in polytrauma patients [3]. However different studies conducted on patients with chest injuries have indicated significant differences in their morbidity and mortality and therefore, further research is essential in order to improve medical care [4, 5].

The purpose of this study was to investigate the epidemiology, characteristics, incidence and management of polytrauma patients with chest injury admitted to our tertiary care facilities’ level I trauma center in order to indicate factors influencing management, possible complications and patient mortality.

## Methods

We performed a retrospective data collection of all polytrauma patients with an ISS ≥ 18 and AIS ≥ 2 in more than one injured body region admitted to the emergency department of our tertiary care center from June 2012 until May 2014. Inclusion criteria were an age above 16 years old, as well as a blunt chest trauma with an AIS_thorax_ ≥ 2. Patients with penetrating chest trauma were excluded from this study. The study was performed after permission was granted by the local ethics committee (#23-12-13(3)).

Detailed information including patient charts were extracted from the hospitals electronic database. Data collection included demographic details, mechanism of injury, details of injuries with scoring based on the Injury Severity Score (ISS) and Abbreviated Injury Scale (AIS).

Management of patients, surgical procedures, length of intensive care unit (ICU) stay, hospital length of stay, as well as injury related complications of these patients were recorded. Factors affecting mortality were evaluated. Because different mechanism of injury were observed among different age intervals, we compared our findings between two age groups of patients: 16–50 years and over 50 years old in order to investigate differences in the treatment and outcome between younger and elderly patients.

### Statistical analysis

Quantitative data is presented as median and range. Statistical analysis was performed using independent samples t-test implemented in the SPSS software package v20.

## Results

A total of 110 patients (82 males) with chest injuries were included in our study. Male to female ratio was 2.79 to 1.00. Median age was 48.5 years (range 17–93 years). Road accidents (car, motorbike and bike accidents) were the most common cause for severe chest injury, followed by fall from a height (25.4%), snow sports accidents (9.2%) and miscellaneous causes (Table 1).

**Table 1:** Mechanism of injury

Mechanism of injury	No. of patients	Percent
Car accidents	32	29.1
Falls	28	25.5
Motorbike accidents	23	20.9
Snow sports	9	8.2
Paragliding	4	3.6
Hot air balloon	3	2.7
Workplace accidents	3	2.7
Climbing	2	1.8
Horse riding	2	1.8
Bike accidents	2	1.8
Skateboard	1	0.9
Base jumping	1	0.9

### Injury pattern

Rib fractures comprised the largest group of thoracic injuries in our series (86.4%) with nearly 75% of them involving three or more ribs. There was a 22.7% incidence of bilateral rib fractures, while in 7 cases flail chest was present (Table 2). The diagnosis of flail chest was made in case of clinical chest wall instability and radiographic confirmation of the diagnosis on chest CT scan (fracture of three or more adjacent ribs in at least two places). The most common associated thoracic injuries were pneumothorax, hemothorax and pulmonary contusion.

**Table 2:** Chest injuries

Chest injuries	No	Percent
Rib Fracture	95	86.4%
> 3 Ribs	68	61.8%
bilateral	25	22.7%
flail chest	7	6.4%
Pneumothorax	65	59.1%
Pulmonary contusion	55	50.0%
Hemothorax	24	21.8%
Clavicle	20	18.9%
Sternal fracture	17	15.5%
Scapula	14	12.7%
Cardiac contusions	11	10.0%
Ruptured diaphragms	2	1.8%
Ruptured aortas	1	0.9%
Tracheobronchial Injury	1	0.9%

As indicated in Table 2, the incidence of pneumothorax was 59.1%, while the incidence of hemothorax was 21.8%. Pulmonary contusions were observed in 55 (50%) patients.

**Fig. 1:**
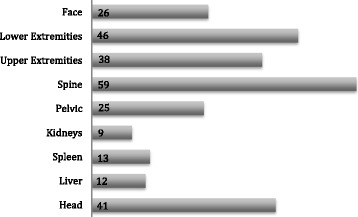
Distribution of Injuries according to body region

The associated injuries of our study population included spine injuries in 59 patients (53.6%), extremity fractures in 84 patients (76.4%) and head trauma in 41 patients (37.3%). The miscellaneous category included pelvic fractures, intra-abdominal injuries and facial trauma as shown in Fig. 1.

### Management

Regarding management, 99 of our thoracic patients with chest trauma were treated conservatively (90%). A chest tube insertion to relieve pneumothorax and/or hemothorax was indicated in 60 of them (54.5%). However observation was possible for patients suffering from small pneumothorax (≤2 cm), without underlying respiratory disease or the need for positive pressure ventilation. 45/60 patients (75%) were treated with chest tube thoracostomy in the emergency department. Median duration of chest tube treatment was 7.5 days (range 1–18 days) depending on the amount of fluid in the drainage chamber, evidence of air leak, and/or expected further surgical operations needing positive pressure ventilation.

Thoracic surgical procedures were performed in 11 cases (10%), including 7 rib stabilizations (6.4%) in patients with a ventilator-dependent flail chest as well as 4 hemothorax evacuations (3.6%) in patients with retained hemothorax not drainable by the indwelling chest tube. Patients requiring thoracic surgery were operated on average 4.5 days after admission. On the contrary, 50 out of a total of 80 (62.5%) non-thoracic surgical operations (orthopedic, neurosurgical, abdominal) had to be carried out within 48h after admission.

### Length of intensive care, mechanical ventilation, and hospital stay

The median hospital length of stay of polytrauma patients with chest injuries was 11 days (range 1–55 days). Seventy-five patients (68.2%) required ICU treatment with a median ICU length of stay of 3 days (range 1–55 days). Intubated patients were referred to the ICU directly, whereas all other patients were transferred to an intermediate care unit (section of the ICU) for further surveillance. Intubation and invasive mechanical ventilation was necessary in 32 patients. The median duration on the ventilator was 5 days (range 1–55 days).

Forty-nine patients (44.5%) were discharged home; all other patients, apart from 6 deaths, were transferred either to other hospitals or to rehabilitation units for further treatment.

### Complications and Mortality

The most common complication was pneumonia (14/110 patients, 12.7%). Sepsis was observed in 6 patients (5.5%), and multi-organ failure in 3 patients (2.7%). Overall 30-day mortality was 5.5% (6/110 patients). All deaths occurred in the context of head trauma or multi-organ failure as a result of hemorrhagic shock. There was no death among the 11 patients who underwent thoracic surgery, nor was there any death directly related to chest injuries

### Abbreviated Injury Severity Score (AIS)

Chest injuries were classified into 4 groups according to the AIS. In our series, 17 patients (15%) sustained a moderate chest injury (AIS_thorax_ = 2), 71 patients (64.5%) a serious chest injury (AIS_thorax_ = 3), while a severe thoracic trauma (AIS_thorax_ = 4) was recorded in 21 patients (19.1%). There was only 1 patient with critical chest trauma (AIS_thorax_ = 5). There were no patients with AIS_thorax_ score of 1 or 6.

The distribution of patients in groups according to the AIS and the results of the statistical analysis between the groups with AIS_thorax_ 2, 3 and 4 are shown in Table 3.

**Table 3:** AIS and clinical characteristics

AIS_thorax_	AIS 2	AIS 3	AIS 4	AIS 5	*p*-value
N (%)	17 (15.5)	71 (64.5)	21 (19.1)	1 (0.9)	
Hospitalization days (median, range)	12 (6–33)	11 (2–55)	11 (4–36)	1	p 0.593
ICU patients n (%)	8 (10.5)	49 (64.5)	18 (85.7)	1 (100)	**p < 0.001**
ICU stay (days) (median, range)	3 (1–20)	3 (1–55)	3.5 (1–24)	1	p 0.34
Intubation days (median, range)	5 (1–6)	5 (1–55)	6 (1–18)	1	p 0.62
Chest tube n (%)	3 (17.6)	37 (52.1)	19 (90.5)	1 (100)	**p < 0.001**
Days chest tube (median, range)	3 (2–4)	7 (2–16)	9 (2–18)	1	p 0.125
Thoracic Surgery	0	6	5	0	
Pneumonia n	2	11	1	0	
Sepsis n	0	3	1	0	
Multi Organ failure	0	4	1	0	
Death n	0	5	0	1	

Significant *p* values are listed in bold

The percentage of patients admitted to the ICU was associated with the severity of the AIS_thorax_ (*p* < 0.001; AIS_thorax_ 2: 10.5% vs. AIS_thorax_ 3: 64.5% vs. AIS_thorax_ 4: 85.7%). Similarly the percentage of patients requiring a chest tube insertion was statistically significantly higher (*p* < 0.001) among the AIS_thorax_ 4 group (90.5%) in comparison with the AIS groups 2 and 3 (17.6% and 52.1% respectively) as expected.

Interestingly the severity of chest trauma did correlate neither with ICU and intubation days nor with the rate of complications and mortality.

### Injury Severity Score (ISS)

The degree of total severity of injury was categorized using the ISS [6–9]. A very severe injury (ISS > 25) was observed in 46 patients (41.9%). According to the calculated ISS, patients were stratified into two groups (ISS: 18–24 and >24)

There was a significant association between the mortality rate and the ISS, as all 6 deaths were in the ISS > 24 group. The median hospitalization time was also significantly longer for this group in comparison to patients with an ISS < 24 (14 days vs. 9 days respectively, *p*  < 0.05).

However, no difference was detected regarding ICU length of stay and ventilator days.

### Comparison between age groups <50 years and >50 years old

In order to investigate the impact of age in management and outcome, patients were divided into two age groups: <50 years and >50 years and the two age groups were compared. Among the older population, rib fractures were more common (*p* < 0.05) and hospital stay was longer (*p* < 0.05). In addition, the elderly underwent surgery significantly earlier (*p* < 0.01) compared to younger patients. Liver injuries as well as injuries of upper extremities were more common in patients younger than 50 years (*p* < 0.05) related to the higher number of motor vehicle accidents observed in this age group.

## Discussion

Although the majority of our patients with blunt chest injury could be treated without surgery (90%), more than half of them required chest tube thoracostomy (54.5%). In our study 75% of the chest tubes were placed in the emergency department, because of the severity of the patients’ general condition, especially in case of tension pneumothorax, traumatic symptomatic pneumothorax and hemothorax, respectively.

As Lesquen et al. [10] mentioned in their review, all traumatic pneumothoraces and symptomatic traumatic hemothoraces should be considered for chest tube insertion in the first 48 h following blunt chest trauma. However, observation is possible for selected patients without respiratory disease or the need for positive pressure ventilation presenting with small unilateral pneumothoraces [11]. Late drainage is usually required in patients with progression of pneumothoraces, hemothoraces or respiratory distress [12, 13].

In accordance with other studies only 10% of our polytrauma patients required surgical treatment for their chest injuries due to unsuccessful non-operative treatment such as non-resolving pneumothorax despite thoracic drainage and/or observation of a persisting air leak in the underwater seal. Condition of a bilateral flail chest with paradoxical movement and/or the need for positive pressure ventilation for more than 48 h was considered an indication for surgery as well [14, 15–17]. This may explain the fact that patients requiring thoracic surgery were operated on average 4.5 days after admission due to our efforts for conservative treatment for patients with stable respiratory function at presentation in the emergency room.

In our patient cohort, 6.4% of the individuals underwent surgical chest wall stabilization. Benefits of a timely chest wall stabilization have been reported in the literature [18]. The severity of chest trauma, based on the AIS, did not correlate with the hospital and ICU length of stay, the time of mechanical ventilation, complications and mortality rates. We explain this finding by the fact that in patients with an AIS_thorax_ of 2 and especially 3 associated injuries such as head and abdominal injuries were much more common than in the group with an AIS_thorax_ of 4. The latter group mainly suffered from the sequelae of an isolated but more severe chest trauma, which is also reflected by a nearly three times higher rate of thoracic surgery compared to patients with an AIS_thorax_ of 3 (23.4% vs. 8.5%). Our data are in line with the findings of Veysi et al. with the exception that mortality was in contrast with our study positively related to AIS_thorax_ [19]. We may assume that an early simple tube thoracostomy in the first 48 h in case of severe pneumothorax, an aggressive pain control including epidural analgesia when necessary (i.e. insufficient pain control with opioids), and intensive chest physiotherapy are the most important factors influencing the outcome after blunt chest trauma [15, 17, 19].

The overall mortality in our cohort reached 5.5%, and was thus significantly lower as compared to previously reported studies [4, 19, 20]. This was maybe owed to the well-developed network of pre-hospital trauma management and the relatively short pre-hospital distances in our country as well as to the hospitals structure of multi-specialist integrated groups and the improved intensive care resuscitation which may result in a survival benefit for trauma patients. Interestingly none of the deaths in our study was attributed to the chest trauma itself. Rather it seems that the severity of an associated head injury significantly correlates with the mortality rate [7, 19, 20], showing the importance of immediate neurosurgical treatment if possible, in order to reduce mortality in polytrauma patients. On the other hand it must also be taken into consideration that that a significant proportion of deaths attributed to critical chest trauma occur in the prehospital setting, although the exact number is difficult to be calculated [21].

Among the elderly population in our study, rib fractures were more common, average hospital stay was longer, and surgery was performed significantly earlier in comparison to the age group < 50. In the elderly the force required to cause rib fractures is less compared to young patients because of osteoporosis, loss of muscle mass, and possible comorbidities [8, 22–24]. Studies have shown that the likelihood of death in case of flail chest increases 132% with every 10 year-increase in age [23]. Furthermore the elderly are more susceptible to pulmonary deterioration and are often affected by other complications such as pneumonia [15]. Therefore early surgical management in this age group may prevent further complications and decrease mortality [18]. In our study the elderly underwent surgery significantly earlier for the reasons described above. Regarding younger patients, fractures of upper extremities are typically more frequent as a result of high-energy trauma, such as motor vehicle accidents.

## Conclusion

Mortality rates in polytrauma patients with blunt chest trauma did not correlate with the severity of chest injury but rather with the severity of associated head injuries in our study cohort. Prompt management of blunt chest trauma with timely chest tube thoracostomy whenever necessary, optimal pain control and chest physiotherapy resulted in good outcome in the majority of patients. Surgical treatment for chest wall stabilization or hemothorax evacuation was only required in a small percentage of polytrauma patients with blunt chest trauma.

## Abbreviations

AIS: Abbreviated injury scale
ISS: Injury severity score
